# Supplementary material for: Anti-human-TIGIT agonistic antibody ameliorates autoimmune diseases by inhibiting Tfh and Tph cells and enhancing Treg cells
Source: Commun Biol. 2023 May 9;6:500. doi: 10.1038/s42003-023-04874-3 (PMC10170076; doi:10.1038/s42003-023-04874-3)
Supplement: Supplementary file 2 — Supplementary information [file 42003_2023_4874_MOESM2_ESM.pdf]

# Supplementary Information

## Anti-human-TIGIT agonistic antibody ameliorates autoimmune diseases by inhibiting Tfh and Tph cells and enhancing Treg cells


Marenori Kojima[1], Katsuya Suzuki[1], Masaru Takeshita[1], Masaki Ohyagi[2], Mana Iizuka[2], Humitsugu Yamane[1], Keiko Koga[3], Taku Kouro[3], Yoshiaki Kassai[3], Tomoki Yoshihara[4], Ryutaro Adachi[4], Kentarou Hashikami[4,5], Yuichiro Ota[1], Keiko Yoshimoto[1], Yuko Kaneko[1], Rimpei Morita[2,6], Akihiko Yoshimura[2], Tsutomu Takeuchi[1]

[1]Division of Rheumatology, Department of Internal Medicine, Keio University School of Medicine, Shinjuku-ku, Tokyo, Japan

[2]Department of Microbiology and Immunology, Keio University School of Medicine, Shinjuku-ku, Tokyo, Japan

[3]Immunology Unit, Pharmaceutical Research Division, Takeda Pharmaceutical Company Limited, Fujisawa City, Kanagawa, Japan

[4]Biomolecular Research Laboratories, Pharmaceutical Research Division, Takeda Pharmaceutical Company Limited, Fujisawa City, Kanagawa, Japan

[5]Present address: Axcelead Drug Discovery Partners, Inc., Fujisawa City, Kanagawa, Japan

[6]Present address: Department of Microbiology and Immunology, Nippon Medical School, Bunkyo-ku, Tokyo, Japan

**Correspondence to:** Professor Tsutomu Takeuchi, Division of Rheumatology, Department of Internal Medicine, Keio University School of Medicine, 35 Shinanomachi, Shinjuku-ku, Tokyo, 160-8582, Japan; tsutake@z5.keio.jp

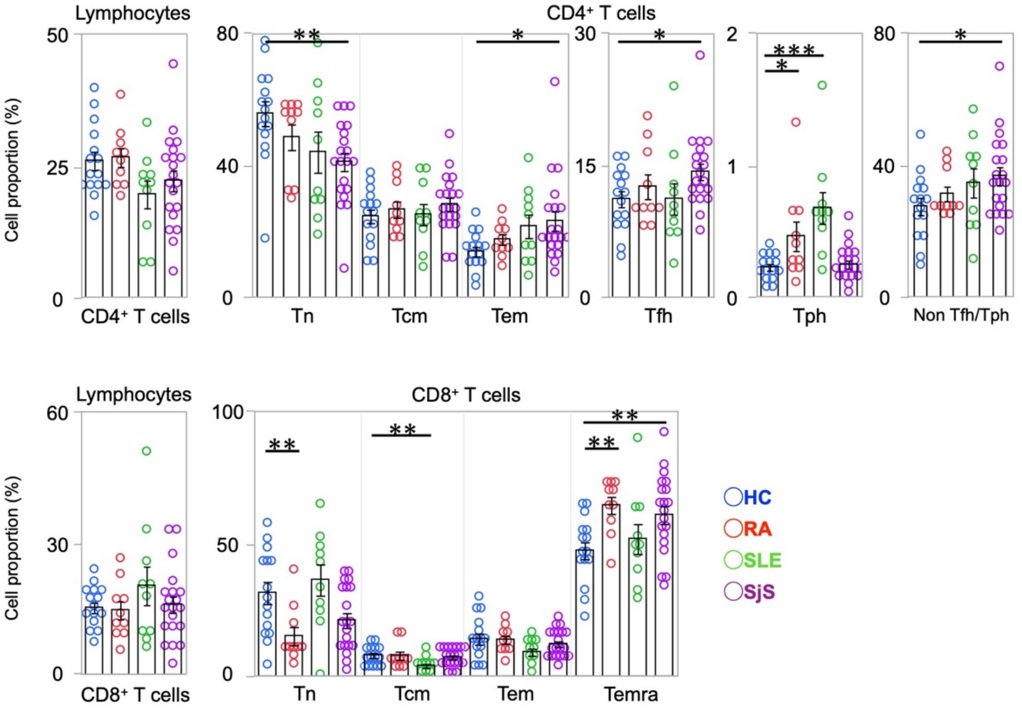


**Supplementary Fig. 1 Some population of effector T cells increased in systemic autoimmune diseases.** PBMCs from 10 untreated RA patients, 10 exacerbated SLE patients, 20 untreated SjS patients and 15 HCs were immunophenotyped. The proportions of $CD4^+$ T cells among lymphocytes and each developmental stage (Tn, Tcm, Tem, Tfh, Tph, non-Tfh/Tph) among the $CD4^+$ T cells from the HCs (blue) and the patients with RA (red), SLE (green) and SjS (purple) are shown. The proportions of $CD8^+$ T cells among the splenocytes and each developmental stage (Tn, Tcm, Tem, Temra) among the $CD8^+$ T cells from the HCs (blue) and the patients with RA (red), SLE (green) and SjS (purple) are shown. Error bars represent the mean ± SEM, and *p* values were determined by the Wilcoxon rank sum test. *$p<0.05$, **$p<0.01$, ***$p<0.001$.

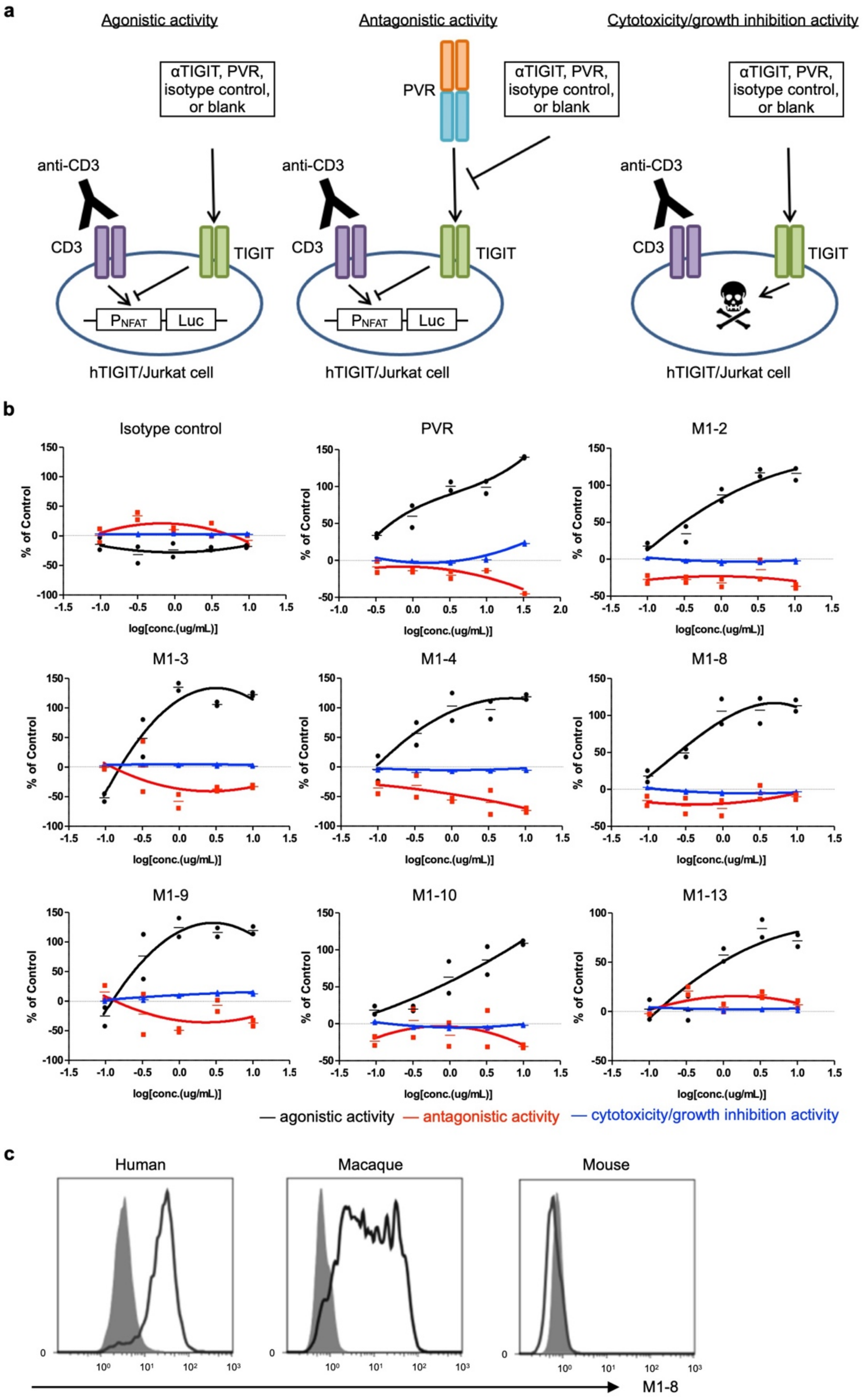


**Supplementary Fig. 2 The developed mAbs possess agonistic activity against TIGIT-expressing cells. a** The schema of the method of agonistic, antagonistic, and

cytotoxicity/growth inhibition activity of mAbs are shown. Finally, the luminescence values were measured using Nano-Glo Luciferase Assay System in agonistic and antagonistic activity and CellTiter-Glo Luminescent Cell Viability Assay System in cytotoxicity/growth inhibition activity. We calculated each activity as follows. The agonist activity (%) (=(1- ((values of sample) – (values of 100% control (PVR 3.3 μg ml-1)))/((values of 0% control (blank)) – (values of 100% control (PVR 3.3 μg ml-1))) × 100)). The antagonist activity (=(((values of sample) – (values of 100% control (blank)))/((values of 0% control (3.3 μg ml-1 PVR)) – (values of 100% control (blank))) × 100)). The cytotoxicity/growth inhibition activity (=(1- ((values of sample) – (values of 0% control (blank)))/(values of 100% control (3.3 μg ml-1 PVR)) – (values of 0% control (blank))) × 100)). **b** Agonistic activity, antagonistic activity, and cytotoxicity/growth inhibition activity of isotype control, PVR, and each mAb are shown. **c** Cross-recognition of M1-8 and human-, macaque-, and mouse-TIGIT is shown.

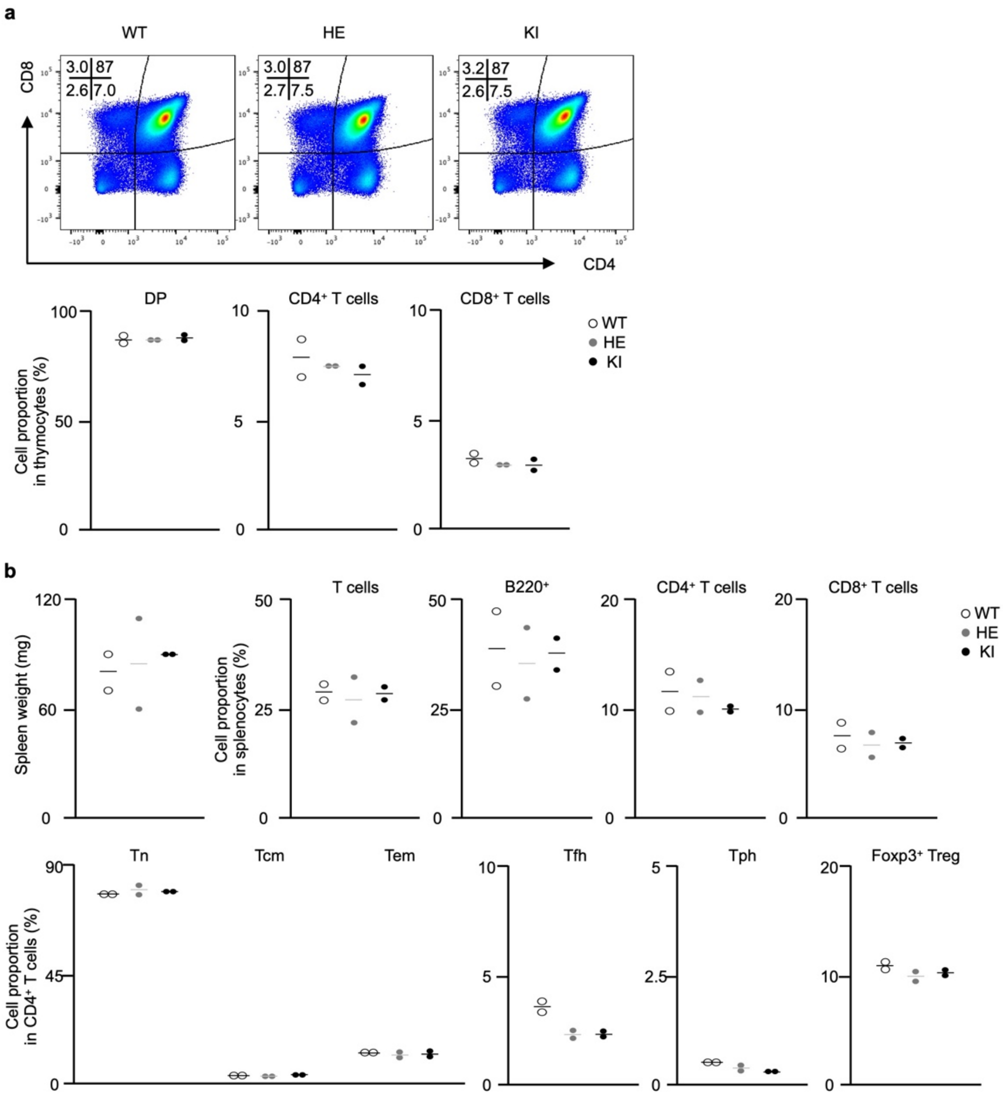


**Supplementary Fig. 3 The proportions of Tfh and Tph cells in knock-in (KI) mice are lower than those in wild-type (WT) mice. a** Flow cytometric analysis of T cell development in 12-week-old female WT (n=2), heterozygous (HE) (n=2) and KI (n=2) mice are checked. Representative flow cytometry plots of thymocytes and the proportions of CD4+, CD8+, and double positive (DP) T cells are shown. **b** The spleen weight in WT (n=2), HE (n=2) and KI (n=2) 10 weeks female mice is shown. The proportions of T, B220$^+$, CD4$^+$ T, CD8$^+$ T cells in splenocytes and Tn, Tcm, Tem, Tfh, Tph, Foxp3$^+$ Treg cells in CD4$^+$ T cells in those mice are shown. Horizontal lines represent the mean of each group.

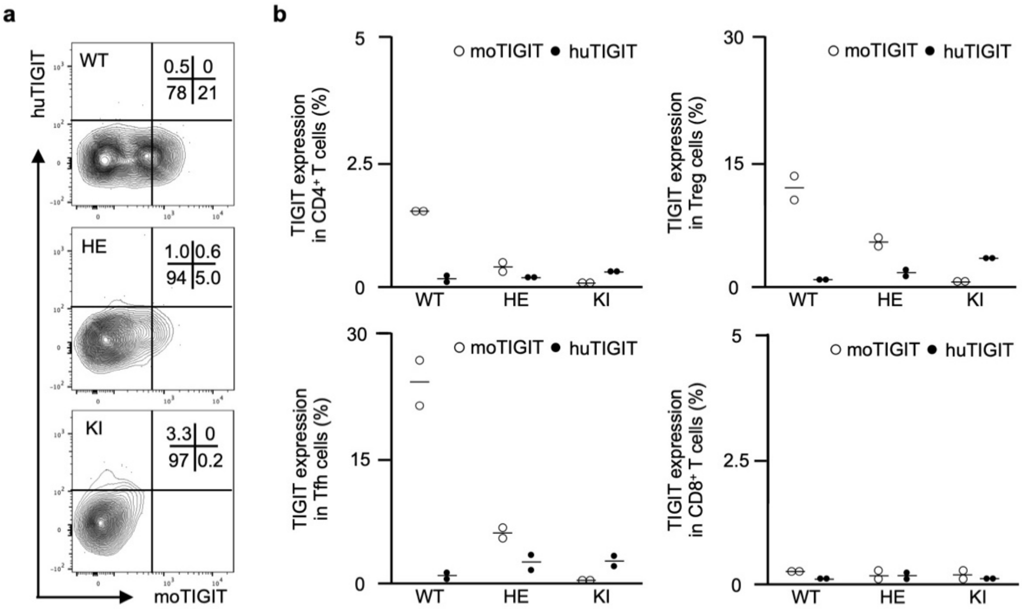


**Supplementary Fig. 4 Human-TIGIT (hu-TIGIT) in knock-in (KI) mice is lower than mouse-TIGIT (mo-TIGIT) in wild-type (WT) mice.** Hu-TIGIT and mo-TIGIT expression of whole $CD4^+$ T, Tfh, Treg, and whole $CD8^+$ T cells in 12-week-old female WT (n=2), heterozygous (HE) (n=2) and KI (n=2) mice are checked, and representative flow cytometry plots of Tfh cells (**a**) and each expression (**b**) are shown. Horizontal lines represent the mean of each group.

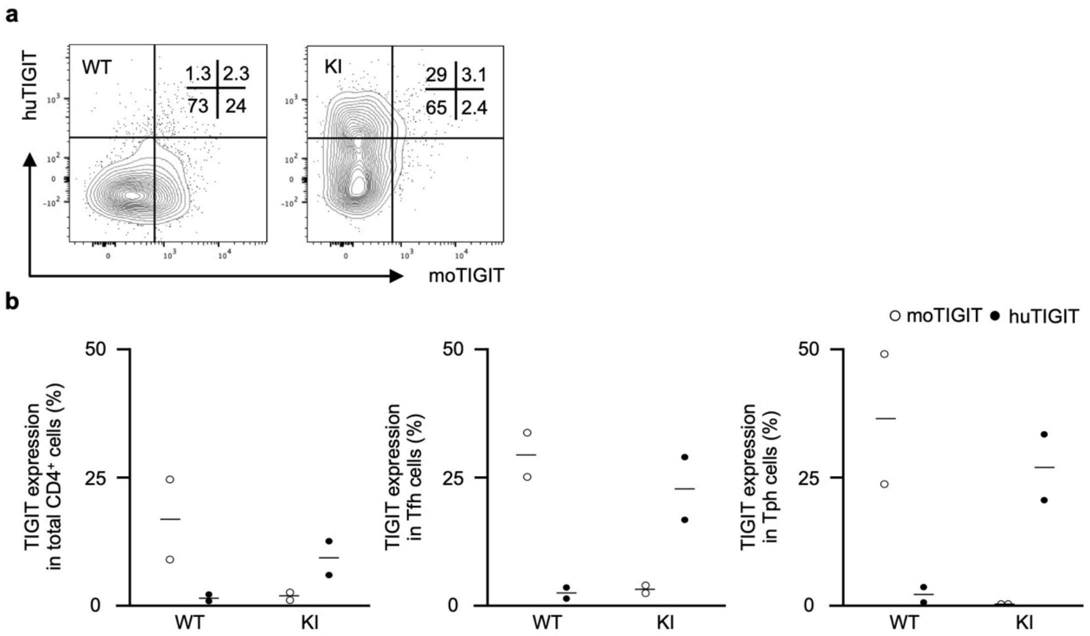


**Supplementary Fig. 5 In the imiquimod (IMQ)-induced lupus model, elevated human TIGIT (hu-TIGIT) expression in knock-in (KI) mice is similar to elevated mouse TIGIT (mo-TIGIT) in wild-type (WT) mice.** 15-week-old WT mice (n=2) and KI mice (n=2) were treated IMQ and induced lupus model. Hu- and mo-TIGIT expression in those mice of whole $CD4^+$ T, Tfh and Tph cells are checked, and representative flow cytometry plots of Tfh cells (**a**) and each expression (**b**) are shown. Horizontal lines represent the mean of each group.

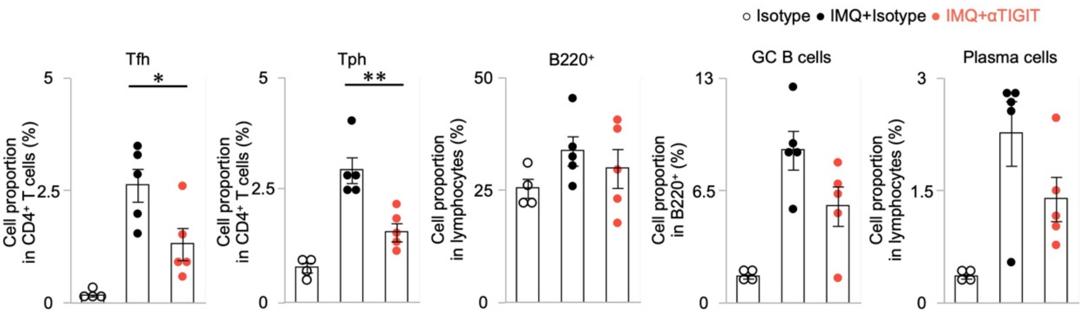


**Supplementary Fig. 6 Anti-human-TIGIT (anti-hu-TIGIT) agonistic mAb (αTIGIT) also suppresses the proliferation of Tfh and Tph cells of lymphocytes in a hu-TIGIT knock-in (KI) imiquimod (IMQ)-induced lupus model in mice.** In the lymph nodes, the proportions of Tfh and Tph cells among $CD4^+$ T cells, $B220^+$ cells and plasma cells ($CD19^{low}$ $CD138^+$) in lymphocytes and germinal centre (GC) B cells ($CD95^+$ $GL7^+$) among $B220^+$ cells are shown. Error bars represent the mean ± SEM, and *p* values were determined using a two-tailed Student's *t*-test. *$p$<0.05, **$p$<0.01, ***$p$<0.001.

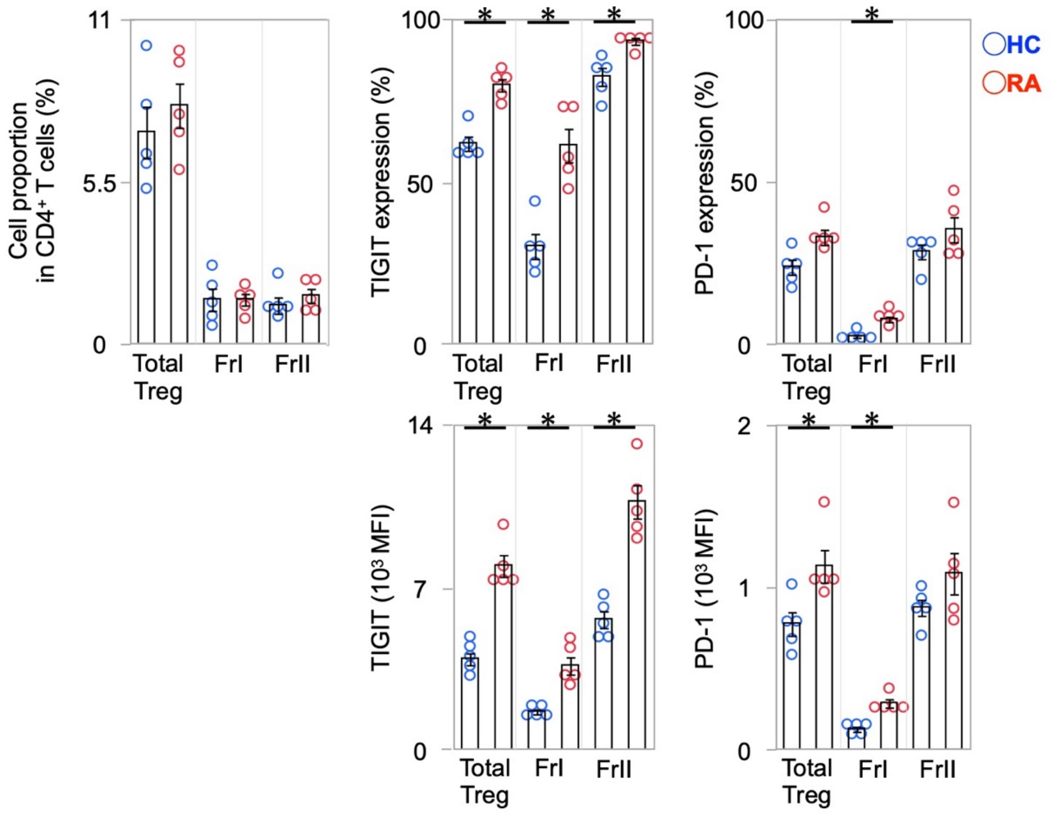


**Supplementary Fig. 7 TIGIT expression of Treg cells is upregulated in patients with RA compared with HCs.** PBMCs from 5 active RA patients and 5 HCs were immunophenotyped. The proportions of each Treg subtype among $CD4^+$ T cells and the proportion and mean fluorescence intensity of TIGIT and PD-1 expression in each Treg subtype from the HCs (blue) and the patients with RA (red) are shown. Error bars represent the mean ± SEM, and *p* values were determined by the Wilcoxon rank sum test. *$p$<0.05, **$p$<0.01, ***$p$<0.001.

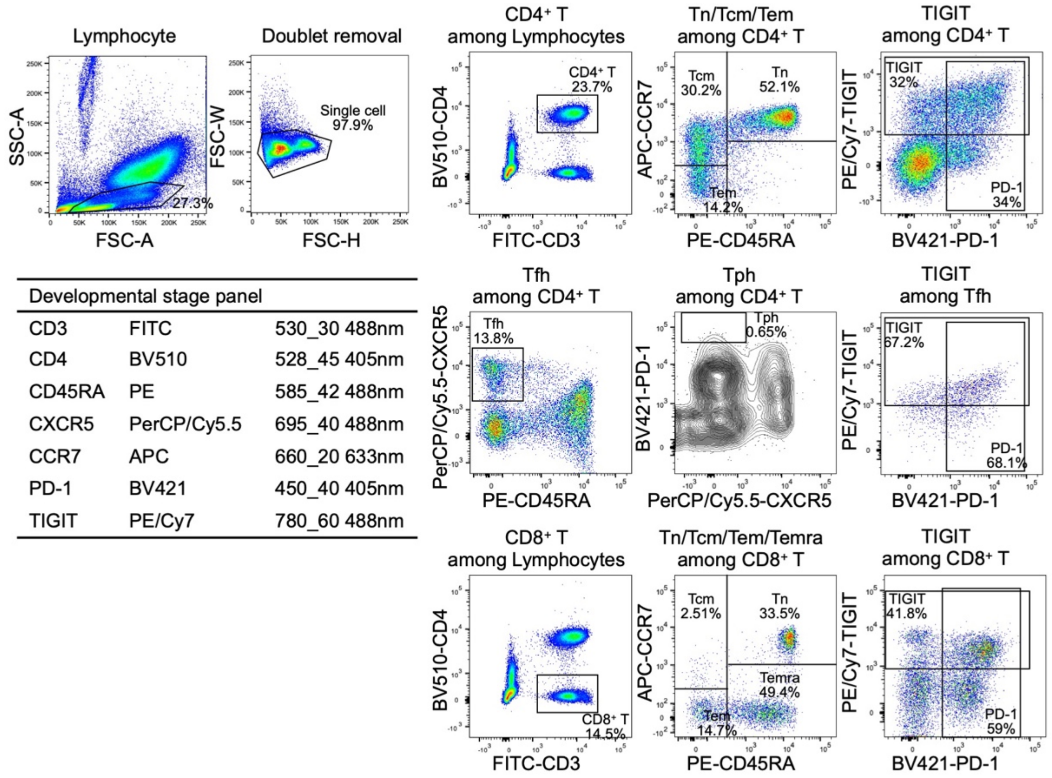


| Developmental stage panel | | |
|---|---|---|
| CD3 | FITC | 530_30 488nm |
| CD4 | BV510 | 528_45 405nm |
| CD45RA | PE | 585_42 488nm |
| CXCR5 | PerCP/Cy5.5 | 695_40 488nm |
| CCR7 | APC | 660_20 633nm |
| PD-1 | BV421 | 450_40 405nm |
| TIGIT | PE/Cy7 | 780_60 488nm |

**Supplementary Fig. 8 Gating strategy.** The gating strategy in immunophenotyping (Fig. 1) is shown. The proportions of Tn, Tcm, Tem, Tfh and Tph cells among CD4$^+$ T cells were defined as shown in a developmental stage panel. The proportions of Tn, Tcm, Tem and Temra cells among CD8$^+$ T cells were also defined as shown in a developmental stage panel. TIGIT and PD-1 expression on total CD4$^+$ and total CD8$^+$ T cells is shown in a developmental stage panel as representative.

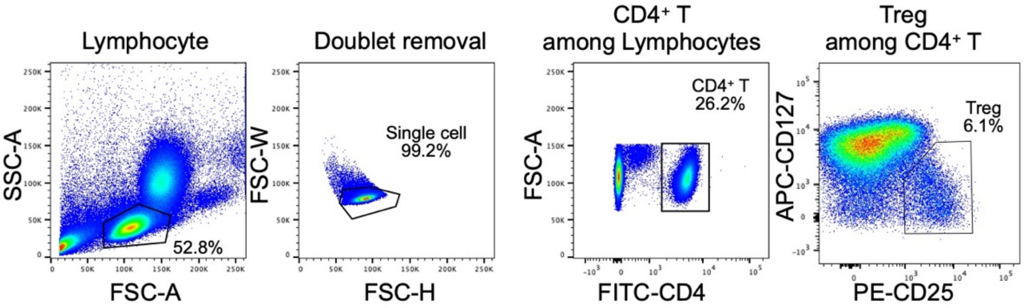


| Developmental stage panel | | |
|---|---|---|
| CD4 | FITC | 530_30 488nm |
| CD25 | PE | 585_42 488nm |
| CD45RA | V450 | 450_40 405nm |
| CD127 | APC | 660_20 633nm |
| PD-1 | APC/Cy7 | 780_60 633nm |
| TIGIT | PE/Cy7 | 780_60 488nm |

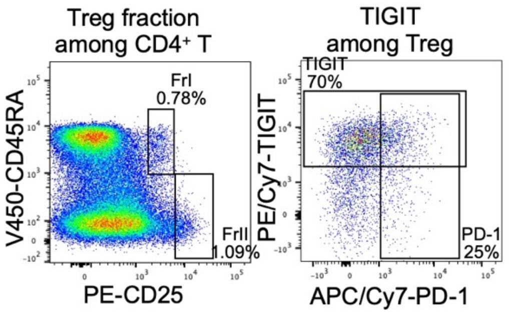


**Supplementary Fig. 9 Gating strategy.** The gating strategy in immunophenotyping (Supplementary Fig. 6) is shown. The proportions of total, FrI and FrII Treg cells among CD4$^+$ T cells were defined as shown in a developmental stage panel. TIGIT and PD-1 expression on total Treg cells is shown in a developmental stage panel as representative.

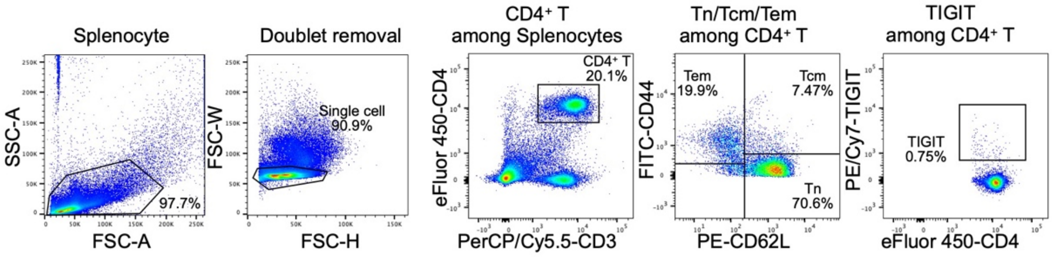


| Developmental stage panel | | |
|---|---|---|
| CD3 | PerCP/Cy5.5 | 695_40 488nm |
| CD4 | eFluor 450 | 450_50 405nm |
| CD44 | FITC | 530_30 488nm |
| CD62L | PE | 585_42 488nm |
| TIGIT | PE/Cy7 | 780_60 488nm |

| Developmental stage panel | | |
|---|---|---|
| CD3 | PerCP/Cy5.5 | 695_40 488nm |
| CD4 | APC/Cy7 | 780_60 633nm |
| CD69 | APC | 660_20 633nm |
| CXCR5 | PE | 585_42 488nm |
| PD-1 | BV421 | 450_40 405nm |
| TIGIT | PE/Cy7 | 780_60 488nm |

| Developmental stage panel | | |
|---|---|---|
| CD19 | FITC | 530_30 488nm |
| B220 | PE | 585_42 488nm |
| CD95 | APC | 660_20 633nm |
| CD138 | BV421 | 450_40 405nm |
| GL7 | PE/Cy7 | 780_60 488nm |

| Developmental stage panel | | |
|---|---|---|
| CD3 | PerCP/Cy5.5 | 695_40 488nm |
| CD4 | eFluor 450 | 450_50 405nm |
| Foxp3 | APC | 660_20 633nm |
| TIGIT | PE/Cy7 | 780_60 488nm |

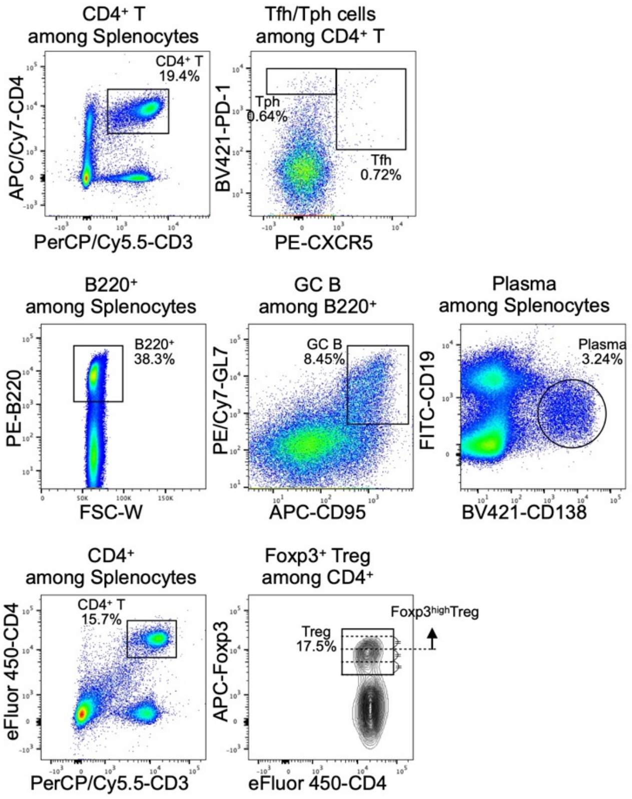


**Supplementary Fig. 10 Gating strategy.** The gating strategy in immunophenotyping (Fig. 3) is shown. The proportions of Tn, Tcm, Tem, Tfh, and Tph and Foxp3$^+$ Treg cells among CD4$^+$ T cells, and B220$^+$ and plasma cells in the spleen, and GC B cells among B220$^+$ cells, and Foxp3$^{high}$ Treg cells were defined as shown in a developmental stage panel. Foxp3$^{high}$ Treg cells were defined as the upper third of the range containing 98% of Foxp3$^+$ Treg cells in CD4$^+$ T cells. TIGIT and PD-1 expression on total CD4$^+$ T cells is shown in a developmental stage panel as representative.

| Developmental stage panel | | |
|---|---|---|
| CD4 | APC/Cy7 | 780_60 633nm |
| CD8a | PE/Cy7 | 780_60 488nm |

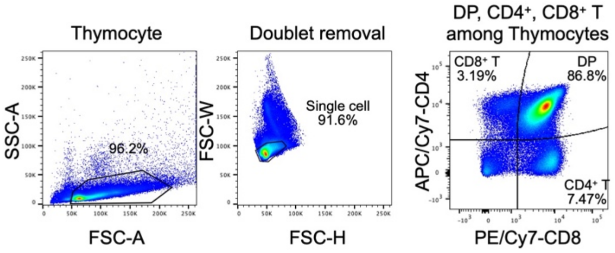


**Supplementary Fig. 11 Gating strategy.** The gating strategy in T cell development (Supplementary Fig. 3) is shown. The proportions of double positive (DP), $CD4^{+}$, and $CD8^{+}$ T cells in thymocytes were defined as shown in a developmental stage panel.

| | HC (n=15) | RA (n=10) | SLE (n=10) | SjS (n=20) |
|---|---|---|---|---|
| Age, years | 38 (35-64) | 62 (41-70) | 35 (25-50) | 55 (44-71) |
| Female, n (%) | 8 (53) | 8 (80) | 8 (80) | 19 (95) |
| DAS28-ESR | | 5.3 (4.5-6.7) | | |
| DAS28-CRP | | 4.6 (3.4-6.3) | | |
| SDAI | | 23 (16-45) | | |
| CDAI | | 20 (16-41) | | |
| RF positive (>15 IU/ml), n (%) | | 7 (70) | | |
| ACPA positive (>5 U/ml), n (%) | | 6 (60) | | |
| CRP, mg/dl | | 0.7 (0.1-4.7) | | |
| ESR, mm/h | | 35 (17-75) | | |
| MMP-3, ng/ml | | 72 (44-323) | | |
| HAQ-DI | | 1.1 (0.6-1.8) | | |
| SLEDAI | | | 15 (11-18) | |
| anti-dsDNA positive (>15 IU/ml), n (%) | | | 5 (50) | |
| ANA ≧ 1:80, n (%) | | | 8 (80) | |
| Low C3 (<65 mg/dl) | | | 3 (30) | |
| Low C4 (<13 mg/dl) | | | 7 (70) | |
| Treatment | | | | |
| None, n (%) | | | 5 (50) | |
| PSL use, n (%) | | | 5 (50) | |
| Immunosuppressant use, n (%) | | | 3 (30) | |
| MMF use, n (%) | | | 1 (10) | |
| ESSDAI | | | | 1.5 (0-5.5) |
| anti-SSA positive, n (%) | | | | 15 (75) |
| anti-SSB positive, n (%) | | | | 5 (25) |
| ANA ≧ 1:80, n (%) | | | | 9 (45) |
| RF positive (>15 IU/ml), n (%) | | | | 10 (50) |
| IgG, mg/dl | | | | 1553 (1332-1922) |

**Supplementary Table 1 Patient background.** The patient background of untreated RA, active SLE and untreated SjS for Figure 1 is shown.

| | RA (n=5) |
|---|---|
| Age, years | 59 (46-78) |
| Female, n (%) | 5 (100) |
| DAS28-ESR | 4.5 (4.1-5.0) |
| DAS28-CRP | 4.7 (3.5-4.9) |
| SDAI | 14 (12-17) |
| CDAI | 12 (11-15) |
| RF positive (>15 IU/ml), n (%) | 5 (100) |
| ACPA positive (>5 U/ml), n (%) | 5 (100) |
| CRP, mg/dl | 0.8 (0.1-2.9) |
| ESR, mm/h | 28 (19-95) |
| MMP-3, ng/ml | 109 (46-519) |
| Treatment | |
| MTX use, n (%) | 3 (60) |
| TCZ use, n (%) | 2 (40) |
| IFX use, n (%) | 1 (20) |

**Supplementary Table 2 Patient background.** The patient background of active RA for Supplementary Figure 6 is shown.

| Anti-human antibodies | Conjugate | Clone name | Lot No. | Supplier |
|---|---|---|---|---|
| CD3 | FITC | UCHT1 | B279208 | BioLegend |
| CD4 | FITC, BV510 | OKT4 | B176551, B277491 | BioLegend |
| CD19 | BV421 | HIB19 | B249392 | BioLegend |
| CD25 | PE | BC96 | B189780 | BioLegend |
| CD27 | BV510 | O323 | B200196 | BioLegend |
| CD38 | FITC | HIT2 | B230710 | BioLegend |
| CD45RA | BV421, PE | HI100 | B262433, B260573 | BioLegend |
| CD127 | APC | A019D5 | B210219 | BioLegend |
| CD138 | APC | MI15 | B238918 | BioLegend |
| CXCR5 | PerCP/Cy5.5 | J252D4 | B26795 | BioLegend |
| CCR7 | AF647 | G043H7 | B255897 | BioLegend |
| PD-1 | APC/Cy7, BV421 | EH12.2H7 | B245235, B268455 | BioLegend |
| TIGIT | PE/Cy7 | A15153G | B264577 | BioLegend |

**Supplementary Table 3 Antibodies and reagents.** The antibodies and regents for human studies are shown.

| Anti-mouse antibodies | Conjugate | Clone name | Lot No. | Supplier |
|---|---|---|---|---|
| CD8a | PE/Cy7 | 53-6.7 | 7215735 | BD Pharmigen |
| CD4 | APC/Cy7 | GK1.5 | B286275 | BioLegend |
| CD44 | FITC | IM7 | B278351 | BioLegend |
| CD62L | PE | MEL-14 | B242685 | BioLegend |
| CD69 | APC | H. 12F3 | B280309 | BioLegend |
| CD95 | APC | SA367H8 | B278865 | BioLegend |
| CD138 | BV421 | 281-2 | B291496 | BioLegend |
| CXCR5 | PE | L138D7 | B309433 | BioLegend |
| PD-1 | BV421 | 29F1A12 | B291919 | BioLegend |
| GL7 | PE/Cy7 | GL7 | B308238 | BioLegend |
| TIGIT | PE | 1G9 | B280925 | BioLegend |
| CD3ε | PE/Cy5 | 145-2C11 | 2143411 | eBioscience |
| CD4 | eFluor 450 | GK1.5 | 4289896 | eBioscience |
| CD19 | FITC | MB19-1 | 4341898 | eBioscience |
| B220 | PE | RA3-6B2 | 4339662 | eBioscience |
| Foxp3 | APC | FJK-16s | 4276015 | eBioscience |

**Supplementary Table 4 Antibodies and reagents.** The antibodies and regents for mouse studies are shown.
