# Supplementary material for: Anti-human-TIGIT agonistic antibody ameliorates autoimmune diseases by inhibiting Tfh and Tph cells and enhancing Treg cells
Source: Commun Biol. 2023 May 9;6:500. doi: 10.1038/s42003-023-04874-3 (PMC10170076; doi:10.1038/s42003-023-04874-3)
Supplement: Supplementary file 3 — Description of Additional Supplementary Files [file 42003_2023_4874_MOESM3_ESM.pdf]

Description of Additional Supplementary Files

File name: Supplementary data

Description: The source data for graphs presented in the article and supplementary information.
