# Supplementary material for: Anti-human-TIGIT agonistic antibody ameliorates autoimmune diseases by inhibiting Tfh and Tph cells and enhancing Treg cells
Source: Commun Biol. 2023 May 9;6:500. doi: 10.1038/s42003-023-04874-3 (PMC10170076; doi:10.1038/s42003-023-04874-3)
Supplement: Supplementary file 5 — nr-reporting-summary [file 42003_2023_4874_MOESM5_ESM.pdf]

Corresponding author(s): Tsutomu Takeuchi
Last updated by author(s): Apr 13, 2023

Reporting Summary

Nature Portfolio wishes to improve the reproducibility of the work that we publish. This form provides structure for consistency and transparency in reporting. For further information on Nature Portfolio policies, see our Editorial Policies and the Editorial Policy Checklist.

Statistics

For all statistical analyses, confirm that the following items are present in the figure legend, table legend, main text, or Methods section.

n/a	Confirmed	
☐	☒	The exact sample size (*n*) for each experimental group/condition, given as a discrete number and unit of measurement
☐	☒	A statement on whether measurements were taken from distinct samples or whether the same sample was measured repeatedly
☐	☒	The statistical test(s) used AND whether they are one- or two-sided<br>*Only common tests should be described solely by name; describe more complex techniques in the Methods section.*
☐	☒	A description of all covariates tested
☐	☒	A description of any assumptions or corrections, such as tests of normality and adjustment for multiple comparisons
☐	☒	A full description of the statistical parameters including central tendency (e.g. means) or other basic estimates (e.g. regression coefficient) AND variation (e.g. standard deviation) or associated estimates of uncertainty (e.g. confidence intervals)
☐	☒	For null hypothesis testing, the test statistic (e.g. *F*, *t*, *r*) with confidence intervals, effect sizes, degrees of freedom and *P* value noted<br>*Give P values as exact values whenever suitable.*
☒	☐	For Bayesian analysis, information on the choice of priors and Markov chain Monte Carlo settings
☒	☐	For hierarchical and complex designs, identification of the appropriate level for tests and full reporting of outcomes
☐	☒	Estimates of effect sizes (e.g. Cohen's *d*, Pearson's *r*), indicating how they were calculated

Our web collection on statistics for biologists contains articles on many of the points above.

Software and code

Policy information about availability of computer code

Data collection	FACSVerse flow cytometer, FACSAria III flow cytometer
Data analysis	Data were analyzed with FLOWJO software version 10.4.2. All statistical analyses were performed with JMP 15 and each test is listed in the respective section.

For manuscripts utilizing custom algorithms or software that are central to the research but not yet described in published literature, software must be made available to editors and reviewers. We strongly encourage code deposition in a community repository (e.g. GitHub). See the Nature Portfolio guidelines for submitting code & software for further information.

Data

Policy information about availability of data

All manuscripts must include a data availability statement. This statement should provide the following information, where applicable:

- Accession codes, unique identifiers, or web links for publicly available datasets
- A description of any restrictions on data availability
- For clinical datasets or third party data, please ensure that the statement adheres to our policy

All data that support our manuscript are accessible from the corresponding authors upon reasonable request.

Field-specific reporting

Please select the one below that is the best fit for your research. If you are not sure, read the appropriate sections before making your selection.

☒ Life sciences ☐ Behavioural & social sciences ☐ Ecological, evolutionary & environmental sciences

For a reference copy of the document with all sections, see nature.com/documents/nr-reporting-summary-flat.pdf

Life sciences study design

All studies must disclose on these points even when the disclosure is negative.

Sample size	There were no statistical tests to determine sample size. The determination of the sample size was based on similar studies in the past (DG Helou, et al. Nature Communications 2020). 4-5 mice per group was selected the most part of the experiments. The number used for each experiment was shown in figure legend.
Data exclusions	No data was excluded.
Replication	In vitro, experiments were performed in duplicate and repeated at least twice independently; in vivo, experiments were also performed twice independently. The same results were obtained for all studies.
Randomization	Generation-, age- and gender- matched mice were randomly assigned in each experiment.
Blinding	Investigators were not blinded to group allocations during the experiment data analysis. Since treatment and experimental analysis could not be separated, blinding of the investigators was not feasible.

Reporting for specific materials, systems and methods

We require information from authors about some types of materials, experimental systems and methods used in many studies. Here, indicate whether each material, system or method listed is relevant to your study. If you are not sure if a list item applies to your research, read the appropriate section before selecting a response.

Materials & experimental systems

n/a	Involved in the study	
☐	☒	Antibodies
☐	☒	Eukaryotic cell lines
☒	☐	Palaeontology and archaeology
☐	☒	Animals and other organisms
☐	☒	Human research participants
☐	☒	Clinical data
☒	☐	Dual use research of concern

Methods

n/a	Involved in the study	
☒	☐	ChIP-seq
☐	☒	Flow cytometry
☒	☐	MRI-based neuroimaging

Antibodies

Antibodies used

Anti-human antibodies
CD3 FITC (clone UCHT1 Biolegend #300406 Lot#B279208)
CD4 FITC, BV510 (clone OKT4 Biolegend #317408 , #317444 Lot#B176551, Lot#B277491)
CD19 BV421 (clone HIB19 Biolegend #302234 Lot#B249392)
CD25 PE (clone BC96 Biolegend #302606 Lot#B189780)
CD27 BV510 (clone O323 Biolegend #302836 Lot#B200196)
CD38 FITC (clone HIT2 Biolegend #303504 Lot#B230710)
CD45RA BV421, PE (clone HI100 Biolegend #304130, #304108 Lot#B262433, Lot#B260573)
CD127 APC (clone A019D5 Biolegend #351316 Lot#B210219)
CD138 APC (clone MI15 Biolegend #356505 Lot#B238918)
CXCR5 PerCP/Cy5.5 (clone J252D4 Biolegend #356910 Lot#B267965)
CCR7 AF647 (clone G043H7 Biolegend #353218 Lot#B255897)
PD-1 APC/Cy7, BV421 (clone EH12.2H7 Biolegend #329921, #329920 Lot#B245235, Lot#B268455)
TIGIT PE/Cy7 (clone A15153G Biolegend #372714 Lot#B264577)
anti-human CD3 (clone HIT3a Biolegend #300332 Lot#B275449)
anti-human CD28 (clone CD28.2 Biolegend #302933 Lot#B281555)
anti-human TIGIT agonistic antibodies and corresponding isotype (Takeda Pharmaceutical Company Limited, patent JP2020-171277)
Anti-mouse antibodies
CD8a PE/Cy7 (clone 53-6.7 BD Pharmigen #552877 Lot#7215735)
CD4 APC/Cy7 (clone GK1.5 Biolegend #100414 Lot#B286275)
CD44 FITC (clone IM7 Biolegend #103005 Lot#B278351)

CD62L PE (clone MEL-14 Biolegend #104407 Lot#B242685)
CD69 APC (clone H. 12F3 Biolegend #104513 Lot#B280309)
CD95 APC (clone SA367H8 Biolegend #152603 Lot#B278865)
CD138 BV421 (clone 281-2 Biolegend #142508 Lot#B291496)
CXCR5 PE (clone L138D7 Biolegend #145503 Lot#B309433)
PD-1 BV421 (clone 29F1A12 Biolegend #135221 Lot#B291919)
GL7 PE/Cy7 (clone GL7 Biolegend #144619 Lot#B308238)
TIGIT PE (clone 1G9 BioLegend #142103 Lot#B280925)
CD3ε PE/Cy5 (clone 145-2C11 eBioscience #15-0031-81 Lot#2143411)
CD4 eFluor450 (clone GK1.5 eBioscience #48-0041-82 Lot#4289896)
CD19 FITC (clone MB19-1 eBioscience #11-0191-82 Lot#4341898)
B220 PE (clone RA3-6B2 eBioscience #12-0452-82 Lot#4339662)
Foxp3 APC (clone FJK-16s eBioscience #17-5773-82 Lot#4276015)

Validation

Describe the validation of each primary antibody for the species and application, noting any validation statements on the manufacturer's website, relevant citations, antibody profiles in online databases, or data provided in the manuscript.

Eukaryotic cell lines

Policy information about cell lines

Cell line source(s)

Jurkat T cells from ATCC

Authentication

Cell lines were not authenticated.

Mycoplasma contamination

Cell lines tested negative for mycoplasma contamination.

Commonly misidentified lines (See ICLAC register)

No commonly misidentified cell lines were used in this study.

Animals and other organisms

Policy information about studies involving animals; ARRIVE guidelines recommended for reporting animal research

Laboratory animals

Human TIGIT knock-in (hu-TIGIT KI) mice with a C57BL/6JJcl background were generated at Takeda Pharmaceutical Company Limited. Wild-type C57BL/6JJcl mice (6–8 weeks) were purchased from Clea Japan, Inc. We bred them and used the same generation of hu-TIGIT KI mice(10-15 weeks old) for each experiment.

Wild animals

This study did not involve wild animals.

Field-collected samples

This study did not involve samples collected from the field.

Ethics oversight

The animals study was performed in accordance with the guidelines for animal care and use approved by Keio University School of Medicine (No. 17071).

Note that full information on the approval of the study protocol must also be provided in the manuscript.

Human research participants

Policy information about studies involving human research participants

Population characteristics

Detailed population characteristics are reported in Supplementary Tables 1 and 2. Age and gender are summarized below:

	HC	RA (1)	SLE	SjS	RA (2)
Age, years (median)	38	62	35	55	59
Female (%)	53	80	80	95	100

There are no other covariates in this study.

Recruitment

The patients who visited Keio University Hospital over a period of 3 years from 2018 to 2020 were recruited. Each patient fulfilled the 2010 American College of Rheumatology/European League Against Rheumatism classification criteria for RA, the 1997 American College of Rheumatology criteria for SLE, and the 2016 American College of Rheumatology/EULAR criteria for SjS. Healthy controls were no personal history of autoimmune diseases and recruited at Keio University.

Ethics oversight

This study was approved by the Institutional Review Board of Keio University School of Medicine (No. 20110258) and conducted in compliance with the tenets of the Declaration of Helsinki. Written informed consent was obtained from all participating individuals.

Note that full information on the approval of the study protocol must also be provided in the manuscript.

Clinical data

Policy information about clinical studies

All manuscripts should comply with the ICMJE guidelines for publication of clinical research and a completed CONSORT checklist must be included with all submissions.

Clinical trial registration	Clinical trial registration was not included in this study.
Study protocol	T cell subsets and the expression of TIGIT and PD-1 on each subset in untreated or active RA, exacerbated SLE or untreated SjS patients was compared with those of healthy controls. The correlation between those expression and each disease activity was analyzed.
Data collection	Among those who visited Keio University Hospital in 2018-2020 were analyzed. Clinical data were collected from medical record.
Outcomes	Not defined in this study because of exploratory study.

Flow Cytometry

Plots

Confirm that:

☒ The axis labels state the marker and fluorochrome used (e.g. CD4-FITC).

☒ The axis scales are clearly visible. Include numbers along axes only for bottom left plot of group (a 'group' is an analysis of identical markers).

☒ All plots are contour plots with outliers or pseudocolor plots.

☒ A numerical value for number of cells or percentage (with statistics) is provided.

Methodology

Sample preparation	For human cells immunophenotyping, 100 µl of blood were collected using a heparin blood collection tube, mixed it with some antibodies, and incubated it for 15 minutes. Then, we added 1.5 ml of FACS Lysing Solution to fix cells and lyse red blood cells, waited 10 minutes, and washed and analyzed.<br>For in vitro study, PBMCs were isolated from the heparinized blood of healthy individuals by using density gradient centrifugation. When needed, CD4+ or CD19+ T cells were isolated by negative or positive selection. The cells were stained with some antibodies and analyzed or sorted.<br>For mouse cells immunophenotyping, the spleen of the mice was chopped into small pieces and lysed with HLB solution through a 40-µm cell strainer. The lymph nodes were passed through a 40-µm cell strainer. After that, each cell stained with some antibodies and analyzed.
Instrument	FACSVerse flow cytometer for data collection, FACSAria III flow cytometer for cell sorting
Software	FLOWJO software version 10.4.2
Cell population abundance	After the cells were sorted, the purity was checked by flow cytometry and it was always above 90%.
Gating strategy	All analyses divided into lymphocyte (FSC-A vs SSC-A) > singlet (FSC-W vs FSC-H) at the beginning. Supplementary Fig. 7 to 9 showed the detailed gating strategy. Basically, cells were divided where they were divided, but isotype controls were used for cells with ambiguous divisions.

☒ Tick this box to confirm that a figure exemplifying the gating strategy is provided in the Supplementary Information.
